# Loss of tau rescues inflammation-mediated neurodegeneration

**DOI:** 10.3389/fnins.2015.00196

**Published:** 2015-06-03

**Authors:** Nicole Maphis, Guixiang Xu, Olga N. Kokiko-Cochran, Astrid E. Cardona, Richard M. Ransohoff, Bruce T. Lamb, Kiran Bhaskar

**Affiliations:** ^1^Department of Molecular Genetics and Microbiology, University of New MexicoAlbuquerque, NM, USA; ^2^Department of Neurosciences, Cleveland Clinic FoundationCleveland, OH, USA; ^3^Department of Biology, University of Texas at San AntonioSan Antonio, TX, USA; ^4^Biogen IdecCambridge, MA, USA

**Keywords:** Alzheimer's disease, tauopathies, tau protein, microglia, neuroinflammation, CX3CR1, microtubule associated protein tau (MAPT), neurodegeneration

## Abstract

Neuroinflammation is one of the neuropathological hallmarks of Alzheimer's disease (AD) and related tauopathies. Activated microglia spatially coexist with microtubule-associated protein tau (*Mapt* or tau)-burdened neurons in the brains of human AD and non-AD tauopathies. Numerous studies have suggested that neuroinflammation precedes tau pathology and that induction or blockage of neuroinflammation via lipopolysaccharide (LPS) or anti-inflammatory compounds (such as FK506) accelerate or block tau pathology, respectively in several animal models of tauopathy. We have previously demonstrated that microglia-mediated neuroinflammation via deficiency of the microglia-specific chemokine (fractalkine) receptor, CX3CR1, promotes tau pathology and neurodegeneration in a mouse model of LPS-induced systemic inflammation. Here, we demonstrate that tau mediates the neurotoxic effects of LPS in *Cx3cr1*^−/−^ mice. First, *Mapt*^+/+^ neurons displayed elevated levels of Annexin V (A5) and TUNEL (markers of neurodegeneration) when co-cultured with LPS-treated *Cx3cr1*^−/−^microglia, which is rescued in *Mapt*^−/−^ neurons. Second, a neuronal population positive for phospho-S199 (AT8) tau in the dentate gyrus is also positive for activated or cleaved caspase (CC3) in the LPS-treated *Cx3cr1*^−/−^ mice. Third, genetic deficiency for tau in *Cx3cr1*^−/−^ mice resulted in reduced microglial activation, altered expression of inflammatory genes and a significant reduction in the number of neurons positive for CC3 compared to *Cx3cr1*^−/−^mice. Finally, *Cx3cr1*^−/−^mice exposed to LPS displayed a lack of inhibition in an open field exploratory behavioral test, which is rescued by tau deficiency. Taken together, our results suggest that pathological alterations in tau mediate inflammation-induced neurotoxicity and that deficiency of *Mapt* is neuroprotective. Thus, therapeutic approaches toward either reducing tau levels or blocking neuroinflammatory pathways may serve as a potential strategy in treating tauopathies.

## Introduction

Neurodegeneration is one of the prominent pathological hallmarks of Alzheimer's disease (AD) and numerous non-AD tauopathies (Lee et al., 2001; Cummings and Cole, 2002). While increasing evidence suggests that pathological modification of microtubule-associated protein, tau (MAPT), and neuroinflammation play a significant role in the neuronal loss observed in tauopathies (Ishizawa and Dickson, 2001; Gerhard et al., 2004, 2006; Wyss-Coray, 2006), the exact mechanism by which these two pathologies result in neurodegeneration remain unclear.

Microglia, the resident inflammatory cells of the central nervous system, monitor the brain for pathological alterations and become activated in most neurodegenerative diseases, including AD and other tauopathies (reviewed in McGeer and McGeer, 2001). Microglial activation can be beneficial or detrimental, contingent upon the context. Activation involves morphological alterations, proliferation, phagocytosis, migration, enhanced expression of cell surface receptors and the production of cytokines (Wyss-Coray, 2006). One way microglia communicate with neurons is via signaling between the neuronally-derived chemokine fractalkine (CX3CL1) and its G-coupled protein receptor, CX3CR1, which is expressed exclusively by microglia within the CNS (Harrison et al., 1998). Unlike other chemokines, CX3CL1 and its cognate receptor CX3CR1, are a unique, one to one ligand-receptor pair known to play an important role in neuroinflammation and neuroprotection. First, CX3CL1 and CX3CR1 signal to each other during chemotaxis (Bazan et al., 1997; Garton et al., 2001). Second, exogenously added CX3CL1 is neuroprotective in models of neuroinflammation (Meucci et al., 1998; Mizuno et al., 2003). Third, a genetic variant with reduced levels of CX3CR1 has been associated with age-related macular degeneration in humans (Combadiere et al., 2007). Fourth, an earlier study from our group demonstrated that disruption of CX3CL1-CX3CR1 signaling in *Cx3cr1*^−/−^ mice (Jung et al., 2000) induces neurotoxicity upon peripheral LPS administration. CX3CR1 deficiency also accelerates neurodegeneration in mouse models of Parkinson's disease (PD) and Amyotrophic Lateral Sclerosis (ALS) (Cardona et al., 2006). Finally, in an earlier study we provided compelling evidence that a single dose of LPS significantly enhanced tau phosphorylation in CX3CR1-deficient mice (Bhaskar et al., 2010). Notably, CX3CR1 deficiency also accelerated tau phosphorylation, aggregation, neuroinflammation as well as cognitive impairment in an hTau mouse model of tauopathy (Bhaskar et al., 2010). Taken together these results suggest that CX3CR1 deficiency enhances neuroinflammation and accelerates tau hyperphosphorylation, which may result in neurodegeneration. However, it is unclear if tau mediates LPS-induced neurodegeneration in the CX3CR1 deficient mice.

Previous studies have suggested that tau can mediate a variety of AD-related phenotypes and that genetic removal of tau is neuroprotective. First, tau-deficient neurons have been shown to be resistant to Aβ-mediated neurotoxicity (Rapoport et al., 2002). Second, genetic deficiency of tau in an AD mouse model improved cognitive function and reduced excitotoxic injury (Roberson et al., 2007; Ittner et al., 2010). Third, Shipton and colleagues recently showed that deficiency of tau protected against Aβ-induced impairment of long-term potentiation in hippocampal slices of wild type mice (Shipton et al., 2011). Fourth, tau deletion has been shown to reduce Aβ-induced defects in axonal transport (Vossel et al., 2010). Finally, a recent study suggested that tau mitigates cognitive impairment induced by type-1 diabetes (Abbondante et al., 2014).

On the other hand, characterization of various lines of tau knockout mice has suggested that tau deficiency is detrimental to the neuronal homeostasis causing axonal dysfunction, neurodegeneration and behavioral impairment (Ahmed et al., 2014; Lei et al., 2014). While these studies suggest that straight tau knockouts may be detrimental in the long-term, it may provide protection against neurotoxic insults such as Aβ. In the current study we demonstrate that deficiency of tau in a CX3CR1-deficient LPS model of neuroinflammation is neuroprotective.

## Materials and methods

### Animals, antibodies, and reagents

*Mapt*^−/−^ (Dawson et al., 2010; De Barreda et al., 2010), and *Cx3cr1*^−/−^ (Jung et al., 2000) (targeted deletion of *Cx3cr1* via insertion of green-fluorescence protein gene into the *Cx3cr1* locus) mice were maintained in the C57BL/6J genetic background and were obtained from the Jackson Laboratory and Dan Littman (HHMI, New York University School of Medicine). *Cx3cr1*^−/−^ mice were crossed to *Mapt*^−/−^ mice to generate *Cx3cr1*^−/−^/*Mapt*^−/−^ mice. The University of New Mexico and Cleveland Clinic Institutional Animal Care and Use Committees (IACUC) approved all animal procedures.

*MAPT antibody:* Mouse monoclonal antibodies—AT8 (Pierce). *Inflammatory markers*: Rabbit polyclonal antibody against ionized calcium binding adaptor molecule 1 (Iba1) (Wako). *Apoptotic antibodies and reagents*: Rabbit monoclonal antibody against CC3 (clone 5A1E; Asp175, Cell Signaling #9664); mouse monoclonal antibody against A5 (EPR3980, Novus Biological/Biotechne); TUNEL kit (Roche; *In Situ* Cell Death Detection Kit, TMR Red; Cat # 12 156 792 910). *Other antibodies:* mouse monoclonal NeuN (Millipore), mouse monoclonal antibody against microtubule-associated protein 2 (MAP2; Sigma).

### Primary cortical neuronal cultures

**Figure 1:**
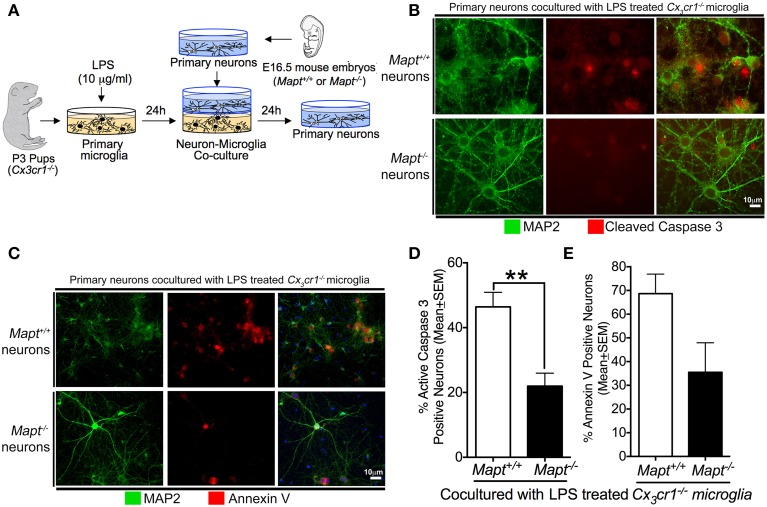
**Loss of tau reduces inflammation-induced expression of CC3 and Annexin V (A5) in primary neurons. (A)** Schematic showing the neuron-microglia co-culture experiments. Primary cortical neurons at 21 days *in vitro* (DIV) (*Mapt*^+/+^ or *Mapt*^−/−^) were co-cultured with LPS (1 μg/ml; 24 h)-stimulated *Cx3cr1*^−/−^ microglia for 24 h. The neurons were fixed and stained for CC3 and A5. **(B,C)** MAP2 positive *Mapt*^−/−^ neurons show reduced CC3 and A5 staining compared to *Mapt*^+/+^ neurons. The blue label in the last panel shows DAPI. Scale bar 10 μm. **(D)** The percentage of CC3^+^MAP2^+^ and A5^+^MAP2^+^ neurons revealed a statistically significant (^**^p < 0.01 for CC3; unpaired *t*-test; *n* = 3 independent experiments; mean ± SEM) reduction for CC3^+^MAP2^+^ for *Mapt*^−/−^ neurons compared to *Mapt*^+/+^ neurons. **(E)** Quantification of A5^+^MAP2^+^ neurons reveals trend toward reduced A5^+^MAP2^+^ immunoreactivity in *Mapt*^−/−^ neurons compared to *Mapt*^+/+^ neurons.

Neuronal cultures were prepared from E16.5 C57BL/6J wild-type (referred to as *Mapt*^+/+^) or *Mapt*^−/−^ mouse embryos as previously described (Bhaskar et al., 2009). All cultures were grown for 21 days *in vitro* (DIV) at 37°C in humidified 5% CO_2_/95% air prior to any treatment. Specifically, primary cortical neurons were seeded onto poly-L-lysine coated coverslips at a density of 1.6 × 10^5^ cells/well in six-well plate for both co-culture and immunofluorescence experiments (Figure 1A).

### Primary microglial culture

Microglial cultures were prepared from post-natal day 3 (P3) pups from *Cx3cr1*^−/−^ genotype as previously described (Saura et al., 2003). Briefly, mixed glial cells were first cultured and grown in T-75 cm^2^ flask seeded at a density of 1.0 × 10^5^−1.2 × 10^5^ cells/cm^2^. After 14 DIV, a differential trypsinization protocol was utilized to remove the astrocytes in the flasks. The pure population of microglia was then seeded at a density of 2 × 10^5^ cells/well in a six-well plate (Figure 1A). Furthermore, the DMEM-F12 media was replaced with Neurobasal media (with no B27 supplement) 24 h prior to the co-culture experiment to match the culture media of primary neurons.

### Neuron-microglia co-cultures

Primary neuronal and microglial cultures were prepared as described above. 21DIV primary cortical neurons grown on a coverslip were placed inside a six-well microplate insert, which has a 0.02 μm Anopore® membrane in the bottom (161395; Nunc). These inserts allow the diffusion of soluble factors into the shared media. The inserts were then placed inside individual wells in a six-well plate, which had LPS (1 μg/ml; for 24 h) (or vehicle) pre-treated *Cx3cr1*^−/−^ or no primary microglia, and incubated for 24 h (Figure 1A). The neurons were fixed in 2% paraformaldehyde (PFA) and processed for immunofluorescence analysis. All the experiments were done in triplicate with independent cultures.

### Lipopolysaccharide and kainic acid administration

Vehicle (Veh, Hank's Balanced Saline Solution) or lipopolysaccharide (LPS; Sigma-Aldrich, USA) (1 mg/kg body weight or b.w, LPS; intraperitoneal or i.p; one dose per day for 3 days or 10 mg/ml single dose for the gene expression and TUNEL analysis) was injected into 2-month-old non-transgenic C57BL/6J (also referred to as *Cx3cr1*^+/+^) and *Cx3cr1*^−/−^/*Mapt*^−/−^ mice. The doses of LPS were selected based on a study, which reported the sub-lethal dosage (<5 mg/kg b.w.) in non-transgenic mice (Qin et al., 2007). 24 h following the final injection, the mice were sacrificed and the right hemisphere was drop-fixed in 4% PFA and processed for neuropathological analysis.

Kainic acid (“KA”; Sigma, K2389) was injected intraperitoneally as previously described (Ghosal and Pimplikar, 2011). Briefly, KA (20 mg/kg b.w; i.p) was administered to 2-month-old C57Bl/6J mice. Animals were sacrificed after 3 days and transcardially perfused with ice-cold phosphate buffer. The brains were removed; the right hemisphere was drop-fixed in 4% PFA and processed for neuropathological analysis.

### Immunocytochemistry

For visualization of CC3 and A5 positive neurons *in vitro*, primary cortical neurons were fixed with 2% PFA, blocked with 5% normal goat serum (NGS) in PBS with 0.4% Triton X-100 for 1 h at room temperature, and incubated overnight at 4°C with the primary antibodies (in blocking buffer) against MAP2 (1:1000) and A5 (1:1000) or MAP2 (1:1000) and CC3 (1:1000). Following 3 washes with PBS at room temperature, cells were incubated with respective secondary antibodies conjugated to Alexa Fluor® 488 and Alexa Fluor® 555 (1:1000; Invitrogen) for 1 h at RT. Cells on coverslips were subsequently washed with PBS and affixed to glass slides via hard-set mounting media with or without DAPI (Vector Laboratories).

#### Quantitative morphometry

For quantitative morphometry, the number of MAP2+A5 and MAP2+CC3 positive neurons was quantified by scoring five random fields per group (*Mapt*^+/+^ or *Mapt*^−/−^) on digital images obtained on a fluorescent microscope at 40x magnification. By assigning the total number of neurons in each field as 100%, double positive neurons in each field were calculated and expressed as % mean ± SEM (*n* = 3 independent co-cultured experiment) of double positive cells per field.

### Immunohistochemistry

Free-floating sections (30 μm thick) were processed for standard immunohistochemistry or immunofluorescence staining. The sections were incubated in 10 mM sodium citrate buffer (pH 6.0) for 10 min at 95°C for antigen retrieval, then washed and blocked in 5% NGS with 0.4% Triton X-100 (blocking buffer) for 1 h at room temperature. Primary antibodies used were at the following dilutions; AT8 at 1:250; Iba1, CC3 and A5 at 1:500; and were incubated overnight at 4°C. Secondary antibodies conjugated to either Alexa Fluor® dyes (1:1000, for immunofluorescence; Invitrogen) or biotin (1:250, for immunohistochemistry; Vector Laboratories) were used. Sections were then either mounted in DAPI Hardset Reagent (for immunofluorescence) or incubated with Avidin:Biotinylated enzyme Complex (ABC reagent, Vector Laboratories; for immunohistochemistry) reagent for 1 h at RT. The immunoreactive signals were revealed by developing sections in SigmaFast® 3,3'-diaminobenzidine (DAB) tablets (Sigma-Aldrich). Bright field and epifluorescence images were acquired using Leica DMR upright fluorescence/bright field microscope. Confocal images were acquired and analyzed with Leica TCS-SP and SP-AOBS upright confocal microscope with Leica confocal software or a Zeiss inverted Meta confocal microscope and Zeiss Zen software.

For double labeling with Terminal deoxynucleotidyl transferase dUTP nick end labeling (TUNEL) and NeuN, first the 30 μm sections were incubated with 2 N HCl for 30 min at 37°C to allow for nuclear permeabilization followed by neutralization with 0.1 M sodium borate buffer (pH 8.6) for 10 min at room temperature. After washing multiple times with PBS, the sections were processed for antigen retrieval as described above. Then the sections were blocked in blocking buffer and processed for TUNEL staining as per manufacturer's instructions (Roche). After washing several times, the sections were processed for double immunofluorescence with a mouse monoclonal antibody against NeuN followed by Alexa Fluor® 488 anti-mouse secondary antibody. The slides were cover-slipped and imaged as described above.

#### Quantitative immunohistochemistry

##### Iba1^+^ area

Iba1 immunoreactive area was quantified using the “*Area Measure*” tool in the Fiji Image J (NIH Software). First the Iba1-immunostained sections (4 random sagittal sections/mouse brain; *n* = 4 mice per genotype) were imaged with a 40x objective and were converted into 8-bit gray scale with a threshold level kept at 89 to create a mask on the Iba1^+^ cells. After applying the threshold mask, the *Analyze > Measure* tool was utilized to measure the total area occupied by Iba1^+^ cells in each image. The percentage of Iba1^+^ area was averaged for three fields per section and all sections per mouse in a genotype. The values expressed are % mean ± SEM per 1 mm^2^ area.

##### CC3^+^ cells in the dentate gyrus (DG)

CC3^+^ cells were manually scored in each digital image (obtained at 40x magnification). Three random fields within the DG were captured per section and three sections per mouse were scored. A total of *n* = 4 per group: *Cx3cr1*^−/−^ (VEH or LPS); *Cx3cr1*^−/−^/*Mapt*^−/−^ (VEH or LPS); non-transgenic (KA) were used for the quantification. Values expressed are mean ± SEM number of CC3+ neurons per 500 μm^2^.

##### TUNEL positive cells

The number of TUNEL positive cells in the granule cell layer of the DG were scored manually per each field (two random fields/section; two sections per genotype) in the max-Z projected confocal digital images taken at 65x magnification. A total of *n* = 4 mice per group: *Cx3cr1*^−/−^ (LPS) and *Cx3cr1*^−/−^/*Mapt*^−/−^ (LPS) were used for quantification. The values expressed are the total number of TUNEL positive cells per μm^2^, mean ± SEM.

### Gene expression analysis

RNA from the hemi-brain (or from co-cultured *Cx3cr1*^−/−^ microglia) was extracted using the Trizol Reagent as described by the manufacturer (Life Technologies). Total RNA (100 ng/μL) was converted to cDNA using the High Capacity cDNA Reverse Transcription kit (Life Technologies, 4368813) and amplified using specific TaqMan probes (Life Technologies, 4331182, see Table 1) and GAPDH was used as a house-keeping gene for normalization, on the StepOnePlus® Life Technologies Real-Time PCR System.

**Table 1:** **List of gene expression assays utilized**.

**Symbol**	**Gene name**	**Assay ID (Life Technologies)**
*CD200R1*	CD200 Receptor 1	Mm00491164_m1
*Tyrobp*	TYRO protein tyrosine kinase binding protein (DAP12)	Mm00449152_m1
*Hmgb1*	High mobility group box 1	Mm00849805_gH
*Hspd1*	Heat Shock Protein 1 (chaperonin, HSP60)	Mm00849835_g1
*IL-1β*	Interleukin 1 beta	Mm00434228_m1
*Trem2*	Triggering receptor expressed on myeloid cells 2	Mm04209424_g1
*IL10RA*	Interleukin 10 receptor alpha	Mm00434151_m1

### Open field test (OFT)

The locomotor behavior of mice was recorded with the open field test. Briefly, each mouse was placed in an open field apparatus equipped with 16 photo beams (San Diego Instruments, San Diego, CA), for a single 15-min session. The time spent by each mouse in either the central or peripheral area of the open field was analyzed.

### Statistical analysis

Data are presented as mean ± SEM, unless otherwise noted, the Student's *t*-test (two-tailed; unpaired) at 95% confidence interval (for two group comparison) or One-Way ANOVA followed by Tukey or Dunnett's *post-hoc* test (for multiple comparisons) was utilized for statistical analysis. Statistical significance was determined at *p* < 0.05.

## Results

### *Mapt*^−/−^ neurons are resistant to microglial neurotoxicity

To determine whether deficiency of tau in the primary cortical neurons will provide resistance against microglial-mediated neurotoxicity, we performed neuron-microglia co-culture experiments. Briefly, *Cx3cr1*^−/−^ primary microglia derived from post-natal day 3 pups were cultured until 14DIV (Saura et al., 2003; Bhaskar et al., 2010). After removal of the astrocyte layer, pure primary microglia were seeded onto plates. Concurrently, primary neurons derived from either non-transgenic or tau knockout (*Mapt*^−/−^) mouse embryos (E16.5) were cultured until 21DIV. For the co-culture experiment, primary microglia were stimulated with 1 μg/ml LPS for 24 h. Then, the primary neurons were co-cultured with the LPS-activated microglia for 24 h. Neurons were then fixed and processed for immunofluorescence analysis for A5 and CC3 to determine the extent of neurodegeneration (Figure 1A). Our results suggest that *Mapt*^+/+^ neurons displayed elevated levels of A5 and CC3 (markers of neurodegeneration) when co-cultured with LPS-treated *Cx3cr1*^−/−^ microglia (Figures 1B,C). Interestingly, *Mapt*^−/−^ neurons cultured in an identical experimental paradigm showed significantly less A5 and CC3 positive neurons (Figures 1B,C). The anti-MAP2 antibody was used to label neurons. Quantification revealed a statistically significant reduction in CC3 positivity in *Mapt*^−/−^ neurons compared to *Mapt*^+/+^ neurons (Figure 1D). Notably, *Mapt*^−/−^ neurons also showed a trend toward reduced A5 positivity compared to *Mapt*^+/+^ neurons. These results suggest that neurons deficient for tau are resistant to CX3CR1-deficient microglial neurotoxicity.

### Genetic deficiency of tau in the *Cx3cr1*^−/−^ mice protects from LPS-induced neurotoxicity

**Figure 2:**
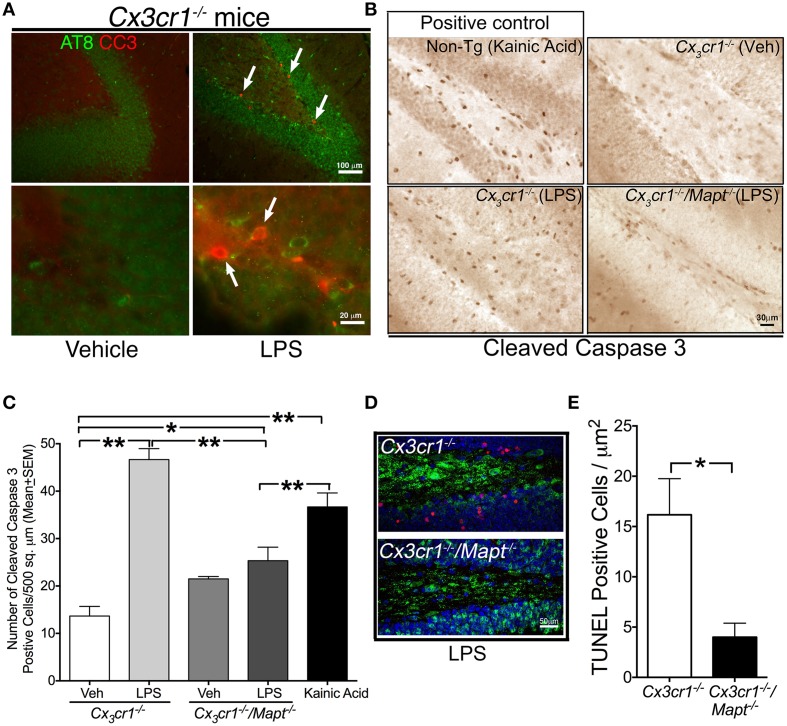
**Tau deficiency reduces LPS-induced neurotoxicity in** ***Cx3cr1*****^−/−^ mice. (A)** Two-month old *Cx3cr1*^−/−^ mice were injected with single dose of vehicle (left panel) or LPS (right panel) (1 mg/kg b.w, daily for 3 days; i.p). Brain sections were double immune-labeled with AT8 (phospho-tau at Ser199) and CC3 antibodies. Presence of AT8 (green) and CC3 (red, arrows) positive cells were detected only in LPS treated samples, primarily in the same sub-population of dentate gyrus. Top panel-low magnification; bottom panel-high magnification. Scale bar 100 μm in top panels and 20 μm in bottom panels. **(B)** The number of cells immunoreactive for CC3 is relatively higher in the DG of 2-month-old *Cx3cr1*^−/−^ mice administered with LPS compared to 2-month-old *Cx3cr1*^−/−^ mice injected with vehicle (Veh). Deficiency of tau in *Cx3cr1*^−/−^/*Mapt*^−/−^ mice shows markedly reduced numbers of CC3^+^ cells following administration of a similar concentration of LPS. As a positive control, 2-month-old non-transgenic mice treated with kainic acid (20 mg/kg b.w, single dose; i.p.) showed a substantial number of CC3^+^ cells in the DG. Scale 30 μm. **(C)** Quantification of CC3^+^ cells reveals a statistically significant (^**^p < 0.01; One-Way ANOVA with Tukey multiple comparison test; *n* = 4 mice per group) increase in CC3^+^ cells in the LPS treated *Cx3cr1*^−/−^ mice compared to the Veh treated mice. Note LPS-treated *Cx3cr1*^−/−^/*Mapt*^−/−^ mice showed significantly less CC3^+^ cells (^**^p < 0.01; One-Way ANOVA with Tukey multiple comparison test; *n* = 4; mean ± SEM) compared to LPS-treated *Cx3cr1*^−/−^ mice or KA-treated mice. Other comparisons are also shown (^*^p < 0.05 or ^**^p < 0.01). **(D,E)** The number of NeuN^+^ neurons (green), which are immunoreactive for TUNEL (red) in the granule cell layer of DG is significantly (^*^p < 0.05; unpaired *t*-test; *n* = 4 mice per genotype; mean ± SEM) higher in the LPS-treated *Cx3cr1*^−/−^ mice compared to the LPS treated *Cx3cr1*^−/−^/*Mapt*^−/−^ mice. Scale bar is 50 μm.

Initial studies from our group examining the role of CX3CR1 in LPS-mediated neurodegeneration (Cardona et al., 2006) and tau hyperphosphorylation (Bhaskar et al., 2010) have been already published. To determine whether tau phosphorylation and neurodegeneration occur in the same susceptible neuronal population within the hippocampus, double immunofluorescence analysis with AT8 and CC3 antibodies were performed in the LPS treated *Cx3cr1*^−/−^ mice. Numerous CC3^+^ cells were observed within the dentate gyrus (DG) of LPS (1 mg/kg b.w, daily for 3 days) injected *Cx3cr1*^−/−^ mice compared to vehicle-injected controls (Figure 2A). Notably, LPS-induced AT8-site tau phosphorylation was observed within the same neuronal population of DG that is also positive for CC3 (Figure 2A), suggesting that LPS administration induces both tau phosphorylation and neurodegeneration within the same susceptible neuronal population of *Cx3cr1*^−/−^ mice.

**Figure 3:**
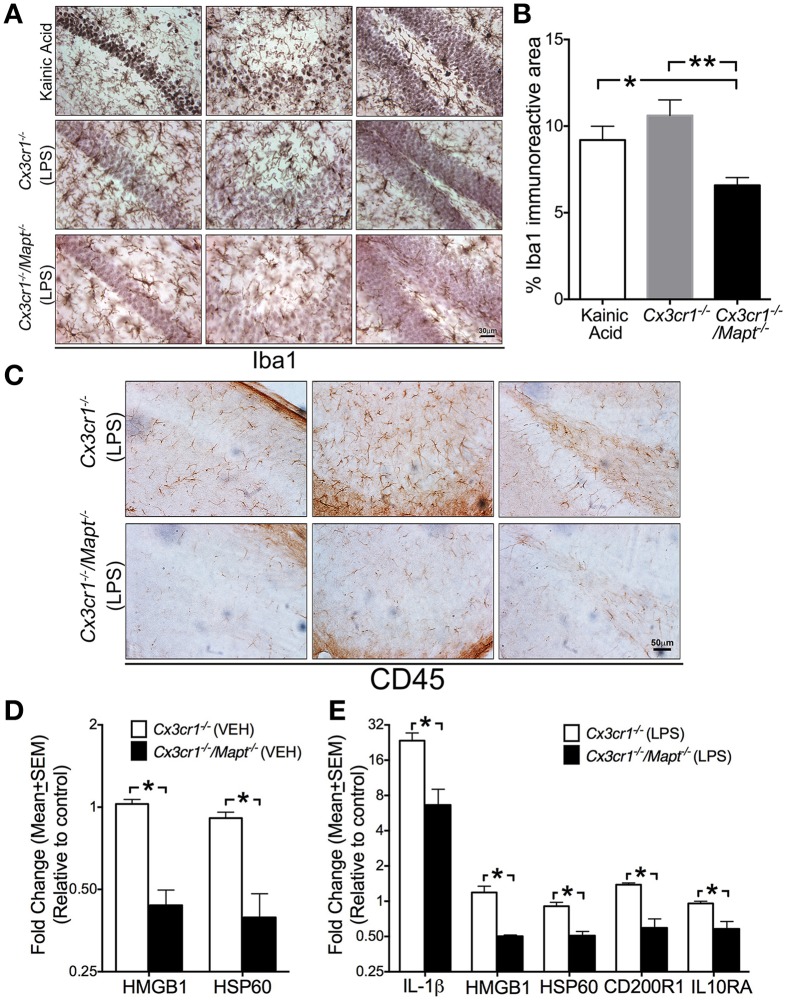
**Tau deficiency in neurons alters microglial immune response. (A,B)** Iba1^+^ microglia display activated morphology in the CA1 (left column in **A**), CA3 (middle column in **A**) and dentate gyrus (right column in **A**) of 2-month-old C57BL/6 injected with KA (20 mg/kg b.w, single dose; i.p) and in 2-month-old *Cx3cr1*^−/−^ mice administered with LPS (1 mg/kg b.w, daily for 3 days; i.p). Note that the 2-month-old *Cx3cr1*^−/−^/*Mapt*^−/−^ mice administered with the same dose of LPS show relatively lower numbers of Iba1^+^ microglia, which also appear less activated. Morphometric quantification reveals a statistically (^*^p < 0.05 vs. KA treated mice; ^**^p < 0.01 vs. LPS treated *Cx3cr1*^−/−^ mice; One-Way ANOVA with Tukey multiple comparison test; *n* = 4 mice/genotype; mean ± SEM) significant decrease in the percentage of Iba1^+^ area in the CA1 subfield in the LPS treated *Cx3cr1*^−/−^/*Mapt*^−/−^ mice compared to LPS treated *Cx3cr1*^−/−^ mice or KA treated controls. Scale 30 μm. **(C)** Reduced CD45^+^ cells in the CA1, CA3, and DG subfields in LPS treated *Cx3cr1*^−/−^/*Mapt*^−/−^ mice compared to LPS treated *Cx3cr1*^−/−^ mice. Scale bar 50 μm. **(D,E)** Quantitative real-time PCR (qRT-PCR) analysis for HMGB1 and HSP60 transcripts (in the vehicle-treated groups; in **D**) and IL-1β, HMGB1, HSP60, CD200R1, and IL10RA (in the LPS-treated groups; in **E**) revealed a significant decrease (normalized to non-transgenics; ^*^p < 0.05; One-Way ANOVA followed by Tukey *post-hoc* test; *n* = 4 mice per group; mean ± SEM) in the inflammatory molecules in the hemibrains of 2-month-old *Cx3cr1*^−/−^/*Mapt*^−/−^ mice compared to *Cx3cr1*^−/−^mice.

To test whether removal of *Mapt* could be neuroprotective in the LPS model *in vivo*, *Mapt*^−/−^ mice were mated with *Cx3cr1*^−/−^ mice to generate *Cx3cr1*^−/−^/*Mapt*^−/−^ mice. Three groups of mice, *Cx3cr1*^+/+^, *Cx3cr1*^−/−^ and *Cx3cr1*^−/−^/*Mapt*^−/−^ were aged to 2 months of age and challenged with LPS (1 mg/kg b.w, daily for 3 days) and examined for various measures of neurodegeneration. Similar to a previously published report from our group (Cardona et al., 2006), the *Cx3cr1*^−/−^ mice injected with LPS displayed numerous CC3 immunoreactive cells within the DG (Figure 2B), while the *Cx3cr1*^−/−^/*Mapt*^−/−^ mice administered with similar dose of LPS displayed a relatively reduced number of CC3 immunoreactive cells (Figure 2B). As a positive control, 2-month-old non-transgenic mice administered with kainic acid (KA, an excitotoxin) showed increased microglial activation (Figure 3A) and CC3 immunoreactivity in the hippocampus (Figure 2B). Upon quantification, the number of CC3^+^ cells in the DG was significantly higher in the LPS injected *Cx3cr1*^−/−^ mice compared to vehicle (Veh) injected mice (Figure 2C), which was similar to non-transgenic mice injected with KA (Figure 2C). Interestingly, the number of CC3^+^ cells in the *Cx3cr1*^−/−^/*Mapt*^−/−^ mice injected with LPS was significantly lower compared to *Cx3cr1*^−/−^ mice injected with an identical dose of LPS (Figure 2C). Next, to confirm that these CC3^+^ cells are indeed degenerating neurons, we performed confocal immunofluorescence analysis using the neuronal marker NeuN and the apoptotic marker TUNEL. The number of NeuN positive neurons showing TUNEL positivity is significantly higher in the molecular layer of DG in the LPS treated *Cx3cr1*^−/−^ mice compared to LPS treated *Cx3cr1*^−/−^/*Mapt*^−/−^ mice (Figure 2D). We performed quantitative morphometry to assess the number of TUNEL and NeuN double positive neurons in *Cx3cr1*^−/−^ and *Cx3cr1*^−/−^/*Mapt*^−/−^ mice injected with LPS. We selected three random sections encompassing three random fields within DG and we scored them manually for double positive cells. The TUNEL positive neurons were significantly lower in the LPS treated *Cx3cr1*^−/−^/*Mapt*^−/−^ mice compared to the LPS treated *Cx3cr1*^−/−^ mice (Figure 2E). Together, these results suggest that genetic deficiency of tau in *Cx3cr1*^−/−^ mice is protective against LPS-induced neurotoxicity *in vivo*.

### Loss of tau provides neuroprotection via altering the microglial immune response

To determine whether the deficiency of tau in neurons affected microglial activation state, we assessed microglial morphology and activation status in three groups of mice: *Cx3cr1*^+/+^, *Cx3cr1*^−/−^, and *Cx3cr1*^−/−^/*Mapt*^−/−^. We identified microglia utilizing microglia-specific Iba1 antibody and an activated microglia/macrophage-specific antibody CD45. The degenerating neurons were identified via CC3 and MAP2 antibodies. Genetic deficiency for *Mapt* in *Cx3cr1*^−/−^ mice resulted in reduced Iba1^+^ microglial number and activation (appearance of swollen cell body with shorter processes) compared to LPS treated *Cx3cr1*^−/−^ mice in the CA1, CA3 and DG of hippocampus (Figure 3A). Notably, as a positive control, Iba1^+^ microglia from KA injected mice displayed a more significantly activated morphology. Interestingly, the CA1 and CA3 neurons displayed pyknotic nuclei with cresyl violet staining (Figure 3A). Quantification of the total Iba1 immunoreactive area within the CA1, CA3, and DG subfields in different brain sections per mouse (*n* = 5 per group) revealed a statistically significant decrease in Iba1^+^ area in LPS treated *Cx3cr1*^−/−^/*Mapt*^−/−^ mice compared to LPS treated *Cx3cr1*^−/−^ mice and KA treated non-transgenic mice (Figure 3B). This suggests that the overall area occupied by microglia is reduced in *Cx3cr1*^−/−^/*Mapt*^−/−^ mice following LPS treatment indicating a less activated status. To further confirm microglial activation, we utilized anti-CD45 antibody. In all three sub-fields of hippocampus (CA1, CA3, and DG), the CD45^+^ cells were more numerous in LPS-treated *Cx3cr1*^−/−^ mice compared to LPS-treated *Cx3cr1*^−/−^/*Mapt*^−/−^ mice (Figure 3C). Taken together markers of microglial inflammatory response (Iba1 and CD45) are significantly reduced because of the deficiency of tau in LPS-treated *Cx3cr1*^−/−^/*Mapt*^−/−^ mice.

**Figure 4:**
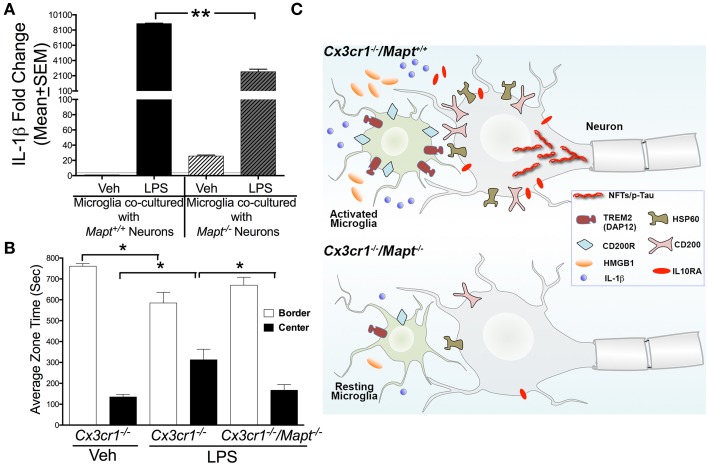
**Tau deficiency reduces microglial-IL-1β secretion** ***in vitro*** **and lack of inhibition in the** ***Cx*****_3_*****cr1*****^−/−^ mice following LPS administration. (A)** qRT-PCR analysis reveal LPS induced pro-IL-1β expression in *Cx3cr1*^−/−^ microglia co-cultured with *Mapt*^+/+^ neurons is significantly (^**^p < 0.01; One-Way ANOVA with Tukey multiple comparison test; *n* = 3 independent co-cultures; mean ± SEM) higher compared to *Cx3cr1*^−/−^ microglia co-cultured with *Mapt*^−/−^ neurons. **(B)** Two-month-old *Cx3cr1*^−/−^ and *Cx3cr1*^−/−^/*Mapt*^−/−^ mice administered with LPS (1 mg/kg b.w, daily for 3 days; i.p) or vehicle were subjected to open-field behavioral test (for 15 min) after 7 days post-LPS treatment. LPS treated *Cx3cr1*^−/−^ mice spent significantly (^*^p < 0.05; One-Way ANOVA with Tukey multiple comparison test; *n* = 4 mice per genotype/group; mean ± SEM) less time in the border, but more time in the center of the maze, compared to vehicle injected *Cx3cr1*^−/−^ mice or LPS injected *Cx3cr1*^−/−^/*Mapt*^−/−^ mice. **(C)** The working model suggests that in *Cx3cr1*^−/−^/*Mapt*^+/+^ mice, LPS induces hyperphosphorylation (p-Tau) and possible aggregation of tau as neurofibrillary tangles (NFTs) due in part to the sustained activation of *Cx3cr1*^−/−^ microglia. This results in upregulation of various inflammatory regulators including neuronally derived ligands (HSP60, CD200, HMGB1), microglial receptors (TREM2/DAP12 and CD200R), IL-1β and IL10RA that are needed to restrain reactive microglia. Despite activation of various negative regulatory pathways of microglia, microglia continue to show significant activation due to the deficiency of the CX3CL1-CX3CR1 communication and LPS. Sustained activation of *Cx3cr1*^−/−^ microglia leads to neurotoxicity possibly mediated by further tau hyperphosphorylation and by the elevated IL-1β signaling. Deficiency of tau in *Cx3cr1*^−/−^/*Mapt*^−/−^ mice show reduced microglial activation, significant reduction in inflammatory mediators including IL-1β and HMGB1, overall decrease in neuron-microglia restraint signaling molecules and thus prevents inflammation-induced, tau-mediated, neurotoxicity.

Next to confirm if the alterations in Iba1^+^/CD45^+^ microglial morphology are a true representation of their activation status, we performed quantitative gene expression analysis on various inflammatory genes within the brains of LPS treated *Cx3cr1*^−/−^ and *Cx3cr1*^−/−^/*Mapt*^−/−^ mice. The genes we selected to investigate are the ones involved in neuron-microglia communications. For example, heat shock protein 60 (HSP60), CD22, CD200 and CD47 are secreted by neurons that interact with their receptors present on microglial surface: Triggering receptor expressed on myeloid cells 2 (TREM2/DAP12), CD45, CD200R1 and CD172a, respectively (reviewed in Ransohoff and Cardona, 2010). The other key molecule secreted by neurons under stress is an alarmin, high mobility group 1 (HMGB1). Among the twenty-one different inflammatory genes assessed, we observed a statistically significant reduction in the basal level expression of HMGB1 and HSP60 in the vehicle injected *Cx3cr1*^−/−^/*Mapt*^−/−^ mice compared to the vehicle injected *Cx3cr1*^−/−^ mice (Table 1; Figure 3D). Remarkably, following LPS treatment the expression levels of interleukin-1β (IL-1β), HMGB1, HSP60, CD200R1 as well as IL10 receptor alpha chain or subunit alpha (IL10RA) were significantly lower in *Cx3cr1*^−/−^/*Mapt*^−/−^ mice compared to *Cx3cr1*^−/−^ mice (Table 1; Figure 3E). Finally to determine if the lack of tau in neurons affects microglial activation state in our cell culture model of LPS-induced neuroinflammation, we assessed the expression of IL-1β in the LPS-treated *Cx3cr1*^−/−^ primary microglia that were co-cultured with *Mapt*^+/+^ or *Mapt*^−/−^ primary cortical neurons. As expected, microglia responded to LPS, showing a significant upregulation of IL-1β expression when they were co-cultured for 24 h with either *Mapt*^+/+^ or *Mapt*^−/−^ primary neurons (Figure 4A). However, microglia co-cultured with *Mapt*^−/−^ primary neurons showed a significantly (four-fold) lower level of IL-1β expression compared to microglia co-cultured with *Mapt*^+/+^ primary neurons (Figure 4A). These results suggest that the presence or absence of tau within neurons could affect overall microglial immune response, specifically pertaining to IL-1β secretion.

### Loss of tau improve anxiety behavior in the *Cx3cr1*^−/−^ mice

To test whether neuroprotective effects elicited by the loss of tau in *Cx3cr1*^−/−^ mice following LPS administration translated into functional effects, we performed various behavioral and cognitive tests. Three groups of mice: 2 month old *Cx3cr1*^−/−^ mice with vehicle or LPS and 2 month old *Cx3cr1*^−/−^/*Mapt*^−/−^ mice with LPS were subjected to a battery of behavioral tests 7 days after the last dose of LPS. While we did not observe any significant alterations in Novel Object Recognition (NOR) test, Morris Water Maze (MWM) or Elevated Plus Maze (EPM) (data not shown), we observed significant alteration in the amount of time the mice spent in the border vs. center of the arena in the Open Field Test (OFT). Notably, vehicle injected *Cx3cr1*^−/−^ mice spent a majority of the time in the border of the OFT arena compared to center, but LPS administered *Cx3cr1*^−/−^ mice spent more time in the center of the arena (Figure 4B). In contrast, LPS injected *Cx3cr1*^−/−^/*Mapt*^−/−^ mice spent more time exploring the border and less in the center, suggesting that LPS administration leads to lack of inhibition in *Cx3cr1*^−/−^ mice, which is rescued by tau deficiency in the *Cx3cr1*^−/−^/*Mapt*^−/−^ mice (Figure 4B).

## Discussion

The present study demonstrates that the loss of tau in neurons provides protection against inflammation-induced neurotoxicity. In the cell culture model of neurons and microglia, pre-treatment of microglia with LPS leads to secretion of cytotoxic factors, which under normal conditions enhances tau hyperphosphorylation and results in neurodegeneration. *Mapt*^−/−^ neurons show significantly reduced levels of apoptotic markers such as CC3 and A5. *In vivo*, systemic administration of LPS causes neurotoxicity in the *Cx3cr1*^−/−^ mice, which is modulated by tau deficiency in the *Cx3cr1*^−/−^/*Mapt*^−/−^ mice.

CX3CL1-CX3CR1 signaling is one of the most well studied pathways involved in the regulation of neuron-microglia communication. Unlike other chemokines, CX3CL1 and its cognate receptor CX3CR1 is a unique, one to one ligand-receptor pair, which is known to play an important role in neuroinflammation and neuroprotection. First, CX3CL1 secreted from neurons and signals to CX3CR1 (on microglia) during chemotaxis (Bazan et al., 1997; Garton et al., 2001). Second, exogenously added CX3CL1 is neuroprotective in models of *in vitro* neuroinflammation (Meucci et al., 1998; Mizuno et al., 2003). Third, a genetic variant with reduced levels of CX3CR1 has been associated with the age-related macular degeneration in humans (Combadiere et al., 2007). Alternatively, several studies suggest that the CX3CL1-CX3CR1 communication may be detrimental in rodent models of acute CNS injury such as stroke (reviewed in Sheridan and Murphy, 2013) and in the model requiring phagocytic activity of microglia [example, clearance of amyloid β (Aβ) plaques Fuhrmann et al., 2010; Lee et al., 2010a].

There are two important aspects of neuron-microglia communication that are relevant for discussion on the present observation. First, the effects reactive *Cx3cr1*^−/−^microglia has on the induction of tau hyperphosphorylation, neuronal survival and homeostasis. Second, the regulation of microglial inflammatory response by the presence of absence of neuronal tau. For the effect of reactive microglia and neuroinflammation on the tau pathology and neuronal survival, several previous studies have demonstrated that neuroinflammation precedes tau pathology in various animal models of tauopathy. First, a large number of inflammatory cells (microglia and astrocytes) and molecules (cytokines, chemokines, complement components, etc…) are present at elevated levels in the AD brain (Eikelenboom et al., 2000; Akiyama et al., 2000; McGeer and McGeer, 2001). Second, patient populations receiving sustained treatment with NSAIDs during mid-life exhibited >50% decreased risk of AD in retrospective studies (McGeer et al., 1996). Third, there is increasing evidence for alterations in inflammatory cells/molecules prior to the development of brain pathologies in several different mouse models of AD (Qiao et al., 2001; Li et al., 2003; Dudal et al., 2004; Kitazawa et al., 2005; Yoshiyama et al., 2007). Fourth, microglial activation precedes tau pathology in a P301S mouse model of tauopathy (Yoshiyama et al., 2007). Fifth, enhancing microglial activation via LPS exacerbated tau pathology in both the 3xTg mouse model of AD and rTg4510 mouse models of tauopathy (Kitazawa et al., 2005; Lee et al., 2010b). Furthermore, blocking microglial activation via an immunosuppressant drug (FK506) attenuated tau pathology and extended the life span in the P301S mouse model of tauopathy (Yoshiyama et al., 2007). Finally, deficiency of CX3CR1 in microglia resulted in enhanced neuroinflammation and neurotoxicity in three different animal models of neurodegeneration (Cardona et al., 2006). Notably, a previous study from our group has suggested that deficiency of CX3CR1 in the hTau mouse model of tauopathy resulted in accelerated tau pathology and cognitive impairment, which is mediated via IL-1β-p38 mitogen activated protein kinase (p38 MAPK) signaling pathway (Bhaskar et al., 2010). It is important to consider that in case of AD, tau hyperphosphorylation may be triggered by Aβ, either directly via the activation of protein kinases such as GSK3β (Ma et al., 2006) or through mediating neuroinflammation, which can then lead to tau pathology (reviewed in Bhaskar and Lamb, 2012). However, the extent of tau phosphorylation directly mediated by Aβ or indirectly via inflammatory alterations needs further study. In another study, altered inflammatory gene expression has been reported to precede classical hallmarks of AD (such as Aβ_40_, Aβ_42_, membrane linked-Aβ or hyperphosphorylated tau), but correlate with increased expression of soluble Aβ oligomers in sporadic AD (Lopez-Gonzalez et al., 2015). Alternatively, in certain animal models of tauopathy, inflammation has been shown to follow, rather precede, tau pathology (reviewed in Dujardin et al., 2015). Together, these studies suggest that microglia-mediated neurotoxic response is a highly complex process and may contribute to the tau pathology and neurodegeneration. However, it is still unclear the type(s) of inflammatory responses that precede and/or follow pathological alterations in tau. In the present study, the presence of CC3 and AT8-site phosphorylated tau within the same neuronal population of the hippocampus suggests that tau hyperphosphorylation may mark susceptible neurons that are destined to die in response to inflammation-induced neurotoxicity. A significant neuroprotection observed in tau negative neurons and in animal models strongly support this possibility, but further work is necessary to confirm how deficiency of tau would prevent the onset/progression of apoptotic and/or other cell-death pathways within neurons in a cell autonomous manner.

The other aspect of the neuron-microglia communication is the regulation of the microglial activation by the presence or absence of tau within neurons. We have observed elevated levels of CX3CL1 in the hTau mouse model of tauopathy at the time of early stage tau pathology (Bhaskar et al., 2010). In a recent study, adeno-associated virus (AAV)-mediated expression of only the soluble form of CX3CL1 resulted in reduced microglial activation and tau pathology in the rTg4510 mouse model of tauopathy (Nash et al., 2013). The primary question still unknown is how loss of neuronal tau could result in reduced neuroinflammation and neurodegeneration? While the CX3CL1-CX3CR1 signaling is one of the major ways neurons communicate with microglia to regulate microglial function, recent studies have suggested that microglia are restrained by neurons based on various additional inhibitory pathways initiated by neurons (reviewed in Ransohoff and Cardona, 2010). Some of the most common pairs of neuron-microglial inhibitory signals are: CD200-CD200 receptor (CD200R) (Hoek et al., 2000; Barclay et al., 2002), CD22-CD45 (Mott et al., 2004), CD172A-CD47(Junker et al., 2009), CX3CL1-CX3CR1 and HSP60–TREM2 (DAP12) (Neumann and Takahashi, 2007; Stefano et al., 2009). Interestingly, an arginine-to-histidine substitution at amino acid 47 (R47H) in the *TREM2* gene has been shown to increase the risk of developing late-onset AD by three-fold (Guerreiro et al., 2013; Jonsson et al., 2013). While these ligand-receptor pairs negatively regulate microglial activation state, it is unclear if one is efficient than the others or if they all play similar role concurrently in dampening microglial activation. A significant reduction in the basal levels of HMGB1 and HSP60 in *Cx3cr1*^−/−^/*Mapt*^−/−^ mice compared to *Cx3cr1*^−/−^ mice suggests that the deletion of tau in neurons alters the levels of the neuron-derived alarmin (HMGB1) and also down-regulates HSP60-TREM2 signaling. At this point, it is unclear whether neurons in *Cx3cr1*^−/−^ mice attempts to keep all the ligand-receptor pairs engaged to restrain reactive microglia, which is subdued in *Cx3cr1*^−/−^/*Mapt*^−/−^ mice due to the lack of pathological tau. On the other hand, a significant reduction in the levels of two important pro-inflammatory molecules (IL-1β and HMGB1) following LPS administration in *Cx3cr1*^−/−^/*Mapt*^−/−^ mice suggests that deficiency of neuronal tau reduces microglial activation via reducing the overall levels of IL-1β and HMGB1 (Figure 4C). While these studies, at present, are correlative, a further characterization is needed to understand if IL-1β and HMGB1 are the only ways to reduce microglial activation in the absence of tau. Recent studies suggested that misfolded tau itself could act like a trigger for microglial activation, resulting in neuroinflammation (Zilka et al., 2009a,b, 2012). Notably, related preliminary studies in our laboratory suggests that when the neuronal tau undergoes pathological modification (such as hyperphosphorylation), they are secreted out to the extracellular space via exosomes. Other groups have also recently demonstrated the secretion of exosome-associated pathological tau (Saman et al., 2012). Such misfolded tau can be internalized by the microglia to induce microglial activation and neuroinflammation (our unpublished study). Based on these studies, we speculate that the absence of tau in *Cx3cr1*^−/−^/*Mapt*^−/−^ mice elicit a reduced microglial activation due to unavailability of pathological tau in the extracellular space in response to LPS-treatment. Together, these studies suggest that tau (when pathologically modified) may directly/indirectly regulate the microglial immune response.

In relation to the effect of inflammatory alterations and behavioral function, several studies have observed a compelling relationship between altered inflammatory responses and behavior functions in various transgenic mice. First, transgenic mice (hsIL-1ra) overexpressing human secreted interleukin-1 receptor antagonist (IL-1ra) displayed higher locomotor scores and less anxiety in the elevated plus maze test (Oprica et al., 2005; Spulber and Schultzberg, 2010). Second, Edaravone, a free radical scavenger, alleviated amyloid toxicity, reduced neuroinflammation and improved OFT behavioral function in APPPS1 mouse model of AD (Jiao et al., 2015). Third, the suppression of human tau transgene with doxycycline in rTg4510 mouse model of tauopathy, rescued behavioral function (OFT, Y-maze and novel object recognition test) and reduced neuroinflammation (Wes et al., 2014). Fourth, AAV-mediated overexpression of fractalkine not only dampens microglial activation, it also reduced neurodegeneration and rescued cognitive deficient in rTg4510 mouse model of tauopathy (Nash et al., 2013). Finally our previous studies suggested that genetically inducing neuroinflammation lead to accelerated working memory impairment in hTau mouse model of tauopathy (Bhaskar et al., 2010). Taken together, these results suggest that altered inflammatory response within the CNS may alter behavioral function.

In summary, our studies suggest that the deficiency of tau within neurons is protective against inflammation-induced neurotoxicity. Furthermore, neurons deficient for tau alter neuron-microglia communication with a net result of reduced inflammatory responses, subdued neuron-microglia molecular machinery, reduced neuroinflammation and increased neuroprotection (Figure 4C). Therefore, future studies should focus on either reducing tau levels or altering the neuron-microglia pathways in the development of future therapies against AD and related tauopathies.

## Author contributions

KB, RR, AC, and BL—Designed the study; NM, GX, OK, and KB—Performed research; RR and AC—Contributed reagents/analytic tools; KB—Wrote the manuscript.

### Conflict of interest statement

The authors declare that the research was conducted in the absence of any commercial or financial relationships that could be construed as a potential conflict of interest.

## Abbreviations

AD: Alzheimer's disease
MAPT: Microtubule Associated Protein Tau
LPS: Lipopolysaccharide
A5: Annexin V
CC3: Cleaved Caspase 3
TUNEL: Terminal deoxynucleotidyl transferase dUTP nick end labeling
PD: Parkinson's disease
ALS: Amyotrophic lateral sclerosis
Aβ: amyloid beta
DIV: Days *in vitro*
DG: Dentate Gyrus
KA: Kainic Acid
Veh: Vehicle
HSP60: Heat shock protein 60
TREM2: Triggering receptor expressed on myeloid cells 2
TYROBP: TYRO protein tyrosine kinase-binding protein
DAP12: DNAX activation protein of 12 kDa
HMGB1: High mobility group 1
IL-1β: Interleukin-1β
MWM: Morris Water Maze
EPM: Elevated Plus Maze
NOR: Novel Object Recognition Test
OFT: Open Field Test
p38 MAPK: p38 mitogen activated protein kinase.
